# Comparison of gastric insufflation using LMA-supreme and I-gel versus tracheal intubation in laparoscopic gynecological surgery by ultrasound: a randomized observational trial

**DOI:** 10.1186/s12871-020-01057-z

**Published:** 2020-06-03

**Authors:** Qiuping Ye, Di Wu, Weiping Fang, Gordon Tin Chun Wong, Yao Lu

**Affiliations:** 1 Department of Anesthesiology, First Affiliated Hospital of Anhui Medical University, No.218 Jixi Road, Hefei, 230022 Anhui Province People’s Republic of China; 2 Department of Anesthesiology, Fuyang Hospital of Anhui Medical University, Fuyang, Anhui Province People’s Republic of China; 3 Department of Anesthesiology, The University of Hong Kong, Hong Kong SAR, People’s Republic of China

**Keywords:** Supraglottic airway devices, Ultrasound, Gastric insufflation, Laparoscopic

## Abstract

**Background:**

The application of bedside ultrasound to evaluate gastric content and volume can assist in determining aspiration risk. Applying positive pressure ventilation via supraglottic airway devices (SAD) can result in a degree of gastric insufflation. This study assessed and compared the antral cross-sectional area (CSA) in patients undergoing laparoscopic gynecological surgery when managed with different SAD.

**Methods:**

One hundred American Society of Anesthesiologists I or II female patients were assessed for inclusion in this study and divided into three groups of different ventilation devices. Patients were randomly allocated into three groups to receive LMA-Supreme (Group S), I-gel (Group I) or tracheal tube (Group T). The primary outcome was the antral cross-sectional area and secondary outcomes included haemodynamic parameters and postoperative morbidity such as sore throat, hoarseness, dry throat, nausea and vomiting.

**Results:**

The antral CSA was not significantly different among three groups before induction (*P* = 0.451), after induction (*P* = 0.456) and at the end of surgery (*P* = 0.195). The haemodynamic variables were significantly higher in the tracheal tube group than in the LMA-Supreme and I-gel groups after insertion (*P* < 0.0001) and after removal (*P* < 0.01). Sore throat was detected in none in the I-gel group compare to two patients (6.7%) in the LMA-Supreme group and fifteen patients (50%) in the tracheal tube group. Hoareness was detected in one (3.3%) in the I-gel group compare to two patients (6.7%) in the LMA-Supreme group and eleven patients (36.7%) in the tracheal tube group.

**Conclusions:**

The SADs do not cause obvious gastric insufflation. Thus, LMA-Supreme and I-gel can be widely used as alternative to endotracheal intubation for the short laparoscopic gynecological surgery.

**Trial registration:**

This trial was registered at the Chinese Clinical Trial Registry (ChiCTR1800018212, data of registration, September 2018).

## Background

Perioperative aspiration of gastric content is a rare but serious anesthetic-related complication which may result in significant morbidity and mortality [1]. Since its recognition back in the 1930s [2], significant measures have been introduce to minimize this complication such as adequate fasting, rapid-sequence induction and the use cuffed tracheal tubes [3]. Supraglottic airway devices (SADs) is now used for procedures requiring positive pressure ventilation that would have been previously managed with an endotracheal tube. Second-generation SADs with gastric channels enables the insertion of a nasogastric tube to either actively or passively vent the stomach. This can potentially minimize gastic insufflation associated with positive pressure ventilation through a suboptimally fitting SAD. For this reason and also because of their versatility and ease of insertion, SADs are increasingly replacing endotracheal tubes [4–6]. Nevertheless, concerns remain that not using cuffed tracheal tubes might result in higher incidence of pulmonary aspiration especially in patients undergoing laparoscopic surgery in the Trendelenburg position. After the creation of a pneumoperitoneum, minute ventilation needs to be increased in order to maintain an acceptable level of arterial partial pressure of carbon dioxide. The abdominal splinting effect of the pnemonperitoneum reduces thoracic compliance and can lead to increase in airway pressures well in excess of 20 cmH_2_O [7]. The rise of airway pressure consequently inducing air leak from the SADs, might excessively insufflate the stomach and cause aspiration of regurgitated contents. However, the degree to which this occurs when compared with endotracheal intubation, or the gastric venting potential of the second generation SADs, have not been quantitatively assessed.

Gastric ultrasound is an emerging point-of-care procedure that has been used to evaluate gastric content and volume in the assessment of perioperative aspiration risk [8]. We therefore performed a prospective randomized clinical trial to compare the degree of gastric insufflation as measured by ultrasound when using endotracheal tube, I-gel or LMA Supreme in laparoscopic gynecological surgery. The primary outcome was the gastric antral cross sectional area as measured by ultrasound. We hypothesized that there will be no significant differences between the devices while the SADs have lower incidence of pharyngeal complications associated with endotracheal intubation.

## Methods

### Trial design and participants

This trial was conducted at the First Affiliated Hospital of Anhui Medical University between September 2018 and March 2019. Patients were prospectively randomized into one of three-group. This trial was registered at the Chinese Clinical Trial Registry (ChiCTR1800018212) on September 5, 2018, and was approved by Institutional Ethics Committee (The First Affiliated Hospital of Anhui Medical University Ethics Committee, PJ2016-08-06, Anhui, China). This study adheres to CONSORT guidelines. Informed consent was obtained from 100 ASA physical status I and II female patients patients aged 18 years or more scheduled to undergo elective laparoscopic gynecological surgery lasting less than 3 h were recruited with ninety patients completing the protocol. We excluded those with preoperative sore throat and / or hoarseness, known risk factors for gastric aspiration, a BMI of 35 or more, Mallampati grade III or 4 and had facial and upper airway abnormalities that would make mask ventilation or tracheal intubation difficult. One patient was excluded because her surgery was canceled. The remaining 99 patients were allocated into three groups (Group S, Group I, Group T) to receive airway management with LMA Supreme, I-gel or tracheal tube respectively, following a computer-generated randomization code.

### Conduct of anesthesia

After arrival in the operating room, all enrolled patients were premedicated with intravenous midazolam 2 mg and standard monitoring (noninvasive assessment of blood pressure, oxygen saturation, pulse oximetry, electrocardiography) was applied. After preoxgenation, patients were induced with etomidate 0.2–0.3 mg/kg, sufentanil 0.5–0.7μg/kg and cis-atracurium 0.2–0.3 mg/kg. Upon the disappearance of the eye lash reflex, the same anesthesiologist applied jaw thrust with the head neutral position. Patients were mask ventilated for 2 min using the Fabius anesthesia machine (Drager, Germany). Controlled ventilation was set to a tidal volume of 8 mLkg^− 1^, frequency of 16 breaths per minute and an inspiratory:expiratory ratio of 1:1.5. The respective airway devices were then inserted accordingly. The cuff of the LMA Supreme was inflated to a pressure of 60 cmH_2_O [9] and the cuff pressure of the endotracheal tube was maintained at 25cmH_2_O [10] by a handheld aneroid pressure gauge. Orogastric tube was also inserted through the supraglottic devices via the gastric channel. Appropriate placement of the airway device was determined by chest expansion, continuous square-wave capnogram, no audible oropharyngeal leak with peak airway pressures (PAWs) of 20 cmH_2_O. If any one of the criteria for satisfactory ventilation was not met, I-gel or LMA Supreme was manipulated by rotating the device in the sagittal plane until the least resistance to bag ventilation was achieved [11]. Patients were then mechanically ventilated by the anesthesia ventilator with a tidal volume of 8 ml/kg and respiratory rate of 12/min and an inspiratory to expiratory ratio (I:E) 1:1.5 and adjusted to maintain the end-tidal CO_2_(EtCO_2_) at around 35–45 mmHg.

Anesthesia was maintained with propofol 4–8 mg/kg·h^− 1^ and remifentanil 6–12 μg/kg·h^− 1^ according to blood pressure and heart rate in the surgery. Muscle relaxation was achieved with cis-atracurium 2–4 mg intermittently. Intra-abdominal pressure (IAP) was at adjusted to around 14 mmHg and Trendelenburg tilt was maintained between 30 and 45° as per surgeons request. At the end of surgery, anaesthesia was discontinued, and the SADs or the tracheal tube were removed when the patient was able to open his or her mouth to command. The cuff was deflated as the devices were removed.

**Fig. 1:**
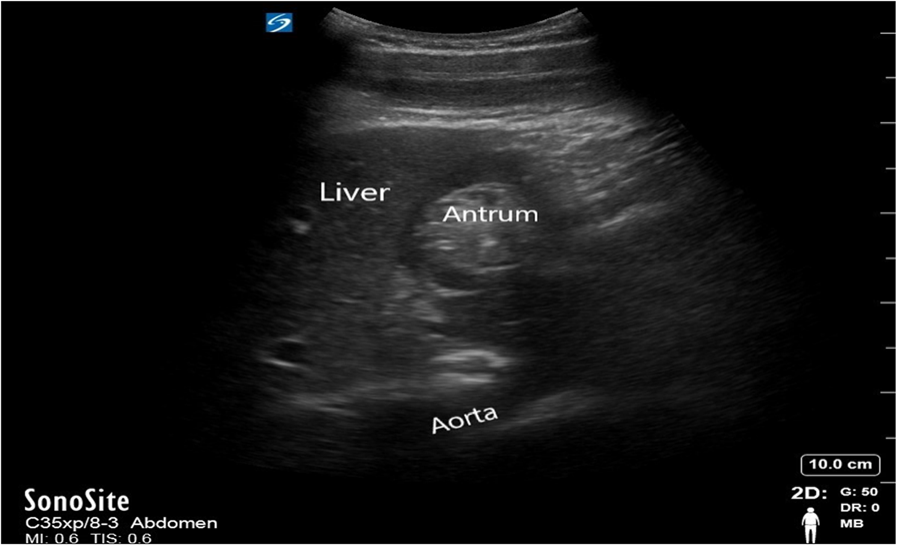
Example of a gastric ultrasonographic image. The antrum was located superficially between the left lobe of the liver anteriorly and the pancreas posteriorly in a sagittal or parasagittal scanning plane in the epigastrium. Important vascular landmarks including the inferior vena cava (IVC) and the superior mesenteric vein was marked the standard scanning plane of the antrum. Antralcross-sectional area (CSA) can be measured by using two perpendicular diameters (antero-posterior diameter and craniocaudal diameter) and the formula of the area of an ellipse

Antral cross-sectional area (CSA) was the primary observed parameter. The stomach was imaged with patient in the supine position by using the low-frequency (2-5 MHz) curved array transducer of a sono ultrasound (FUJIFLIM SonoSite Inc. USA) machine. The antrum was located superficially between the left lobe of the liver anteriorly and the pancreas posteriorly in a sagittal or parasagittal scanning plane in the epigastrium. Important vascular landmarks including the inferior vena cava (IVC) and the superior mesenteric vein was marked the standard scanning plane of the antrum [12]. Antral CSA can be measured by using two perpendicular diameters (antero-posterior diameter and craniocaudal diameter) and the formula of the area of an ellipse (Fig. 1). Antral CSA was noted before induction, immediately after induction and the end of surgery.

Surgical data of the patients included: patient characteristics (age, weight, height, calculate BMI, American Society of Anesthesiologists class), airway assessment (Mallampati class, thyromental distance) and operative details (time). Haemodynamic variables including systolic blood pressure (SBP), diastolic blood pressure (DBP) and heart rate (HR) were recorded in the moment of before the induction (baseline T_1_), the moment of before intubation immediately (T_2_), the moment of finishing intubation immediately (T_3_), the moment of after operating (T_4_) and the moment of after extubation immediately (T_5_). For each patient, the following complications occurring during insertion, maintenance and removal were noted: aspiration or regurgitation; coughing or retching; and blood staining of the SAD or the endotracheal tube. In the event of intraoperative failure of the SAD, the need to intubate was recorded.

Patients were interviewed 24 h after leaving the recovery room and were asked about the presence of a sore throat, dry throat, hoarseness, nausea and/or vomiting. Nausea symptoms were graded using a visual analogue scale (VAS) by the patient as nill, mild, moderate or severe [13]. A blinded trained observer collected the data during the study.

### Statistical analysis

Sample size was performed using SPSS software based on our preliminary study showing an increased mean antral CSA for patients in LMA Supreme group and I-gel group (405 ± 105 and 400 ± 95, respectively) compared with patients in endotracheal tube group (340 ± 94) at the end of the surgery. To detect differences in antral CSA at the end of the surgery with an SD of 95, the sample size was calculated as 29 per group at a power of 80% and a two-tailed α-error of 5%. We enrolled 100 patients in total to countervail potential dropouts.

Statistical analysis was conducted using the SPSS 17.0 software (SPSS Inc., Chicago, USA). Studied data were expressed as mean ± SD, or in frequencies and percentages when appropriate. We compared normally distributed continuous variables among the groups using one-way ANOVA, and used a least significant difference (LSD) procedure for post hoc comparisons. Mann-Whitney U tests were applied for intergroup comparisons when a significant difference was detected between the groups. Categorical variables were compared using chi-squared test. All comparisons were two sides and a *P* value of less than 0.05 was required to exclude the null hypothesis.

## Results

**Fig. 2:**
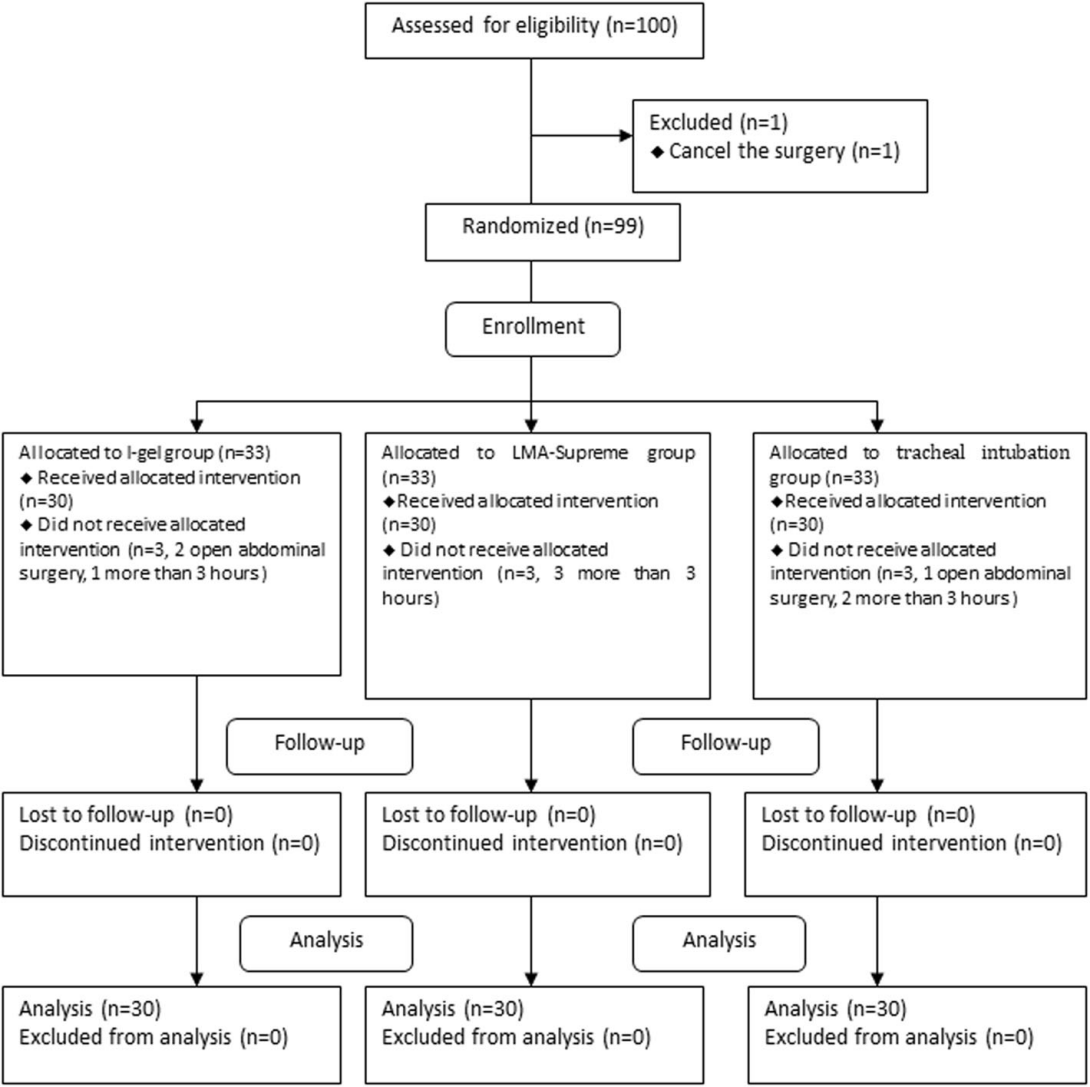
Consort flow chart that outline patients assignment and treatment protocols. Patients were allocated into three groups (Group S, Group I, Group T) to receive airway management with LMA Supreme, I-gel or tracheal tube respectively, following a computer-generated randomization code

A total of 100 patients were enrolled for this study. There was one exclusion for cancellation (Fig. 2). There were allocated as follows: 33 patients included in the I-gel group, 33 in the LMA Supreme group and 33 in the tracheal intubation group. There were no significant differences in patient characteristics among the groups (Table 1). The antral CSAs were not significantly different among three groups in the moment of before induction (*P* = 0.451), the moment of after induction (*P* = 0.456) and the moment of after the surgery (*P* = 0.195). There was no difference in the antral cross-sectional area among Group S, Group I and Group T (*P* = 0.814; Table 2).

**Table 1:** Baseline Characteristics

Variable	Group I	Group S	Group T	P value
Age (year)	39.57 (12.03)	42.43 (11.75)	43.40 (12.07)	0.174
Weight (kg)	59.07 (6.29)	59.37(7.10)	63.13(7.93)	0.054
Height (cm)	159.20 (5.60)	159.83 (5.15)	160.93 (4.43)	0.413
BMI (kg m^−2^)	23.38 (2.95)	23.29 (3.05)	24.36 (2.68)	0.289
ASA physical status				0.475
I	23 (76.6%)	25 (83.3%)	21 (70%)	
II	7 (23.4%)	5 (16.7%)	9 (30%)	
Mallampati score				0.600
I	1 (3.3%)	3 (10%)	1 (3.3%)	
II	18 (60%)	15 (50%)	20 (66.7%)	
III	11 (36.7%)	12 (40%)	9 (30%)	
Thyromental distance (cm)	7.67 (0.83)	7.78 (0.70)	7.48 (0.55)	0.940
Duration of anesthesia (min)	127.13 (38.78)	129.47 (37.71)	130.40 (34.90)	0.921
Duration of pneumoperitoneum (min)	92.20 (39.99)	95.83 (37.95)	95.20 (32.81)	0.257

Data are expressed as number (percentage) or mean (SD). ASA, American Society of Anesthesiologists. Group I, Group I-gel; Group S, Group LMA Supreme; Group T, Group tracheal tube

**Table 2:** Antral cross-sectional area

	Group I	Group S	Group T	P value
S1	330.41 ± 105.21	348.74 ± 151.05	370.09 ± 101.39	0.451
S2	362.20 ± 106.14	391.86 ± 152.00	401.59 ± 115.95	0.456
S3	401.13 ± 108.52	410.32 ± 153.57	355.74 ± 103.70	0.195

Data are expressed as mean ± SD. S1, antral CSA was noted before induction; S2, antral CSA was noted after induction; S3, antral CSA was noted after surgery. Group I, Group I-gel; Group S, Group LMA Supreme; Group T, Group tracheal tube

This data are analyzed by Repeated Measures F = 0.206, P = 0.814

The SBP, DBP and HR are summarized for each time point in Table 3. There were no significant differences in SBP, DBP and HR among the three groups in the moment of T1, T2 and T4. The SBP, DBP and HR were significantly higher in the tracheal tube group than in the LMA-S and I-gel groups after insertion(T3) (*P* < 0.0001). SBP and HR were significantly higher in the tracheal tube group than in the LMA-S and I-gel groups after their removal(T5) (*P* < 0.01). Compared with T2 and T4, the SBP, DBP and HR of the tracheal tube group were significantly increased in T3 and T5.

**Table 3:** Haemodynamic data

	Group	T_1_	T_2_	T_3_	T_4_	T_5_
SBP	Group I	124.17 ± 18.35	108.47 ± 11.12	109.23 ± 14.07^c^	115.13 ± 15.96	117.43 ± 14.40 ^c^
mmHg	Group S	125.40 ± 12.09	114.10 ± 11.58	111.10 ± 13.28 ^c^	116.03 ± 13.10	114.43 ± 11.20 ^c^
	Group T	124.97 ± 15.97	106.77 ± 13.40	125.60 ± 16.02^a, c^	113.17 ± 10.95	124.37 ± 11.52^b, c^
DBP	Group I	72.10 ± 10.76	64.60 ± 8.17	62.30 ± 10.33 ^c^	68.03 ± 10.31	67.93 ± 9.15
mmHg	Group S	72.53 ± 7.66	64.90 ± 7.24	64.40 ± 9.13 ^c^	67.30 ± 9.61	64.80 ± 7.30
	Group T	69.13 ± 9.42	60.97 ± 8.64	73.83 ± 11.15^a, c^	64.27 ± 7.87	71.07 ± 7.57^b^
HR	Group I	77.40 ± 7.87	64.53 ± 6.45	65.70 ± 8.19 ^c^	72.20 ± 10.23	70.63 ± 9.02^b, c^
bpm	Group S	78.07 ± 14.73	66.87 ± 8.35	66.80 ± 10.90 ^c^	73.73 ± 10.72	74.00 ± 9.91 ^c^
	Group T	79.27 ± 13.70	65.40 ± 11.20	85.57 ± 13.57^a, c^	71.17 ± 7.27	85.73 ± 6.96^b, c^

Data are expressed as mean ± SD. Compare with T_2_, ^a^P < 0.05, Compare with T_4_, ^b^P < 0.05, Compare between three groups, ^c^P < 0.05. Group I, Group I-gel; Group S, Group LMA Supreme; Group T, Group tracheal tube

Data regarding sore throat, hoarseness, dry throat, nausea and vomiting are summarized in Table 4. There was no difference in dry throat, nausea and vomiting but sore throat and hoarseness were statistically different between groups (*P* < 0.0001). None of the three groups of patients had a serious postoperative complications of reflux aspiration.

**Table 4:** Postoperative morbidity data(%)

Complication event	Group I	Group S	Group T	P value
sore throat	0 (0.0)	2 (6.7)	15 (50)	0.000
hoarseness	1 (3.3)	2 (6.7)	11 (36.7)	0.000
dry throat	25 (83.3)	25 (83.3)	24 (80)	0.927
Nausea				0.135
No nausea	21 (70)	25 (83.3)	18 (60)	
Mild nausea	4 (13.3)	3 (10)	7 (23.4)	
Moderate nausea	5 (16.7)	2 (6.7)	3 (10)	
Severe nausea	0 (0)	0 (0.0)	2 (6.6)	
vomiting	7 (23.4)	2 (6.7)	5 (16.7)	0.200

Data are number/patients with data (percentage). Group I, Group I-gel; Group S, Group LMA Supreme; Group T, Group tracheal tube

## Discussion

I-gel is a relatively new kind of SADs, which is made of medical grade thermoplastic elastomer and designed according to anatomical characteristics. The soft noninflatable cuff is well sealed around perilaryngeal framework, and effectively isolates laryngeal opening from oropharyngeal opening. The lack of inflatable cuff might result in lower incidence of sore throat [14, 15]. The buccal stabilizer of this device equipped with an airway tube and a separate gastric channel, tends to adapt its shape to the patient’s oropharyngeal curvature [6, 16]. LMA-Supreme has an inflatable cuff and its design includes a more rigid structure, a larger size, a drain tube and the presence of gills to push the epiglottis upward. The LMA Supreme has been shown to be safe and efficacious. Ultrasound were used for antral diameter measurement, and they show up to be a powerful tool in hands of airway managers, given they offer the opportunity to evaluate the patient for difficult intubation [17], and fasting status [18], they can be used for tube position control [19], to support cricothyrotomy and tracheostomy [20] and also to assess effective positioning of SADs [21].

In this prospective randomized trial, we found that the antral cross-sectional areas in different points of time were similar between the LMA-S, I-gel and endotracheal tube. However, the blood pressure and heart rate of patients in the tracheal tube group increased significantly after intubation and after extubation. Postoperative sore throat and hoarseness were higher in endotracheal tube group. Other postoperative signs of poor tolerance of the devices (dry throat, nausea and vomiting) were similar between groups.

Our study provides additional information to evaluate the gastric insufflations in choosing the ventilation devices in laparoscopic gynecological surgery. The tracheal tube is the “gold standard” for avoiding gastric aspiration and reflux in general anesthesia. However, the use of supraglottic airway devices has a series of advantages, such as lower fluctuations in hemodynamics, easier insertion than tracheal tube and a significant reduction in the incidence of sore throat and hoarseness and so on. In recent years, SADs have been widely used in various clinical operations [22–24]. The primary limitation of the supraglottic airway devices is that it does not reliably protect the lungs from regurgitated stomach contents [4]. Both CO_2_ pneumoperitoneum and Trendelenburg’s position lead to elevated airway pressure, and this would theoretically increase gastric air content. Should this occur, this may potentially obsure the visual field of the surgical site hence increases the difficulty of surgery, and may increases the incidence of postoperative nausea and vomiting, and reduces the satisfaction of postoperative recovery period. Our study showed that this is unlikely to occur in this surgical population. Further the blood pressure and heart rate were more stable and a lower incidence of sore throat and hoarseness after mask insertion or after mask removal. Research report the SADs may act as a barrier at the level of the upper oesophageal sphincter if they are correctly positioned [25]. The incidence of aspiration with the SADs has been estimated at 0.02%, which is similar to tracheal intubation in elective patients [26].

Our study has several limitations. First of all, this is only the single center. Thus, a multicenter study would be better to further determine this hypothesis. Sencondly, the patient population was not overweight and of reasonable general health. Thirdly, the devices are from different operators and I-gel gastric channel is much smaller than Supreme. Lastly, we did not assess for amount of air that was passes onto the small bowel that may have caused postoperative abdominal discomfort.

## Conclusion

This study shows that both LMA-Supreme and I-gel were effective for controlled ventilation after the creation of pneumoperitoneum in the Trendelenburg position. They have potential advantages of stable hemodynamic parameters and lower incidence of sore throat and hoarseness compared to tracheal tube, also do not cause obvious gastric insufflations. Thus LMA-Supreme and I-gel can be widely used as alternative to endotracheal intubation for the short laparoscopic gynecological surgery.

## Data Availability

The datasets analysed during the current study are available from the corresponding author upon reasonable request.

## Abbreviations

SAD: supraglottic airway device
CSA: cross-sectional area
PAWs: peak airway pressures
EtCO_2_: end-tidal CO_2_
IAP: intra-abdominal pressure
IVC: inferior vena cava
SPO2: blood oxygen saturation leve
VAS: visual acuity scores
ASA: American Society of Anesthesiologists
SBP: Systolic blood pressure
DBP: diastolic blood pressure
HR: heart rate
